# Optimal search patterns in honeybee orientation flights are robust against emerging infectious diseases

**DOI:** 10.1038/srep32612

**Published:** 2016-09-12

**Authors:** Stephan Wolf, Elizabeth Nicholls, Andrew M. Reynolds, Patricia Wells, Ka S. Lim, Robert J. Paxton, Juliet L. Osborne

**Affiliations:** 1 Rothamsted Research, Harpenden, UK; 2 School of Biological & Chemical Sciences, Queen Mary University of London, UK; 3 School of Life Sciences, University of Sussex, Brighton, UK; 4 School of Biological Sciences, Queen’s University Belfast, Belfast, UK; 5 Institute of Biology, Martin-Luther-Universität Halle-Wittenberg, Germany; 6 Environment and Sustainability Institute, Penryn, University of Exeter, UK

## Abstract

Lévy flights are scale-free (fractal) search patterns found in a wide range of animals. They can be an advantageous strategy promoting high encounter rates with rare cues that may indicate prey items, mating partners or navigational landmarks. The robustness of this behavioural strategy to ubiquitous threats to animal performance, such as pathogens, remains poorly understood. Using honeybees radar-tracked during their orientation flights in a novel landscape, we assess for the first time how two emerging infectious diseases (*Nosema* sp. and the *Varroa*-associated Deformed wing virus (DWV)) affect bees’ *behavioural performance* and *search strategy. Nosema* infection, unlike DWV, affected the spatial scale of orientation flights, causing significantly shorter and more compact flights. However, in stark contrast to disease-dependent temporal fractals, we find the same prevalence of optimal Lévy flight characteristics (μ ≈ 2) in both healthy and infected bees. We discuss the ecological and evolutionary implications of these surprising insights, arguing that Lévy search patterns are an emergent property of fundamental characteristics of neuronal and sensory components of the decision-making process, making them robust against diverse physiological effects of pathogen infection and possibly other stressors.

Most animals benefit from behavioural decision-making based on information about their current environment^1^. Any movement pattern that promotes the efficient acquisition of informative cues can thus be assumed advantageous^2^. This is particularly true for central place foragers exploring novel or transient unpredictable environments, where efficient sampling of informative cues is crucial for their ability to return to the nest, den or colony. In such cases, movement patterns resembling Lévy flights can be advantageous because these patterns of movement curtail needless resampling of terrain when searching blindly with limited or no information about the landscape^3^,^4^,^5^,^6^. Lévy flights alternate clusters of many short steps (bouts of unidirectional flight) with longer steps between them, creating fractal movement patterns that have no characteristic scale. The hallmark of a Lévy flight is a step-length distribution with a heavy power-law tail: *P(l)* ~ *l*^*−μ*^. Accordingly, Lévy flights seem to govern probabilistic search movements in a wide and diverse range of terrestrial and aquatic organisms including basking sharks, bony fish, turtles, jellyfish, honeybees, fruit flies, the wandering albatross, *E. coli*, human T cells, human hunter-gatherers, and have even been observed in trace fossils – the oldest records of animal movement patterns^7^,^8^,^9^,^10^,^11^,^12^,^13^,^14^.

While much attention has been paid to how Lévy flight-based searches are affected by environmental context, prey density or distribution e.g.^7^,^10^,^15^,^16^,^17^,^18^,^19^, there is very limited information on how internal physiological factors may affect an animal’s search strategy^20^. This is surprising since analysis of fractal characteristics of the temporal structure of behavioural sequences (e.g. time allocation to foraging, resting, grooming), which are also associated with optimized interactions with the environment, reveal that both environmental and internal physiological variables strongly affect behavioural optimization. Fractal complexity in behavioural sequence structure was found to increase with both complexity^21^ and novelty of the environment^22^. While an increased motivation to forage may lead to more complex sequential patterns^23^, most forms of physiological stress (social disharmony, pregnancy, intoxication, pathogen load) entailed an overall reduction of behavioural complexity^24^,^25^,^26^, resulting in more stereotypical and suboptimal behavioural sequences. For example, in Spanish ibex, the sequential time allocation pattern between feeding and vigilance was significantly less complex in both pregnant and parasite-infected females^24^. Health status was also a major factor affecting time spent moving and foraging in Japanese macaques^21^,^24^. However, the effects of physiological stress, such as that induced by pathogen infection, on the *spatial* search patterns in animals (e.g. foraging, mate-search) has been highlighted as a major open question in the understanding of how universal laws of optimal searches arise and shape animal behaviour^20^,^27^.

We address for the first time the effects of pathogen infection on optimized scale-free search behaviour, using honeybees (*Apis mellifera*) as a model organism. Honeybees are central-place foragers exploring vast areas of up to 300 km^2^ in search of floral resources^28^,^29^. Their navigation relies on learned and memorized landmarks; though there is a debate about exactly how bees utilize landmark information^30^,^31^,^32^,^33^,^34^. When they first leave the hive, honeybee workers have to efficiently acquire a sufficient set of informative landmarks to allow them to navigate back to the hive. They do so by performing successive and spatially increasing orientation flights around the colony^35^,^36^,^37^. Lacking any *a priori* information about the position of landmark cues, their orientation flights represent probabilistic searches and as such can be expected to have Lévy flight characteristics. Indeed, orientation flights in both honeybees and bumblebees have been found to follow a Lévy distributed optimal search strategy^37^,^38^.

The possibility to reliably record optimized search behaviour using harmonic radar^16^,^38^ in combination with the current call for a better understanding of the individual and colony-level effects of the numerous native and exotic emerging pathogens threatening an economically valuable livestock animal and pollinator^39^,^40^ makes honeybees an ideal model system to study the optimization of movement patterns in the context of pathology. Here we focus on two types of emergent honeybee disease with fundamentally different modes of action: *Nosema ceranae*, a gut parasite acquired only by adult bees and mainly interfering with the digestive system and energetics^41^,^42^ (henceforth, *Nosema*), and a virus-complex (Deformed wing virus (DWV) and a variant of DWV termed DWV genotype B or Varroa destructor virus (VDV), henceforth DWV), which is transmitted by the parasitic mite *Varroa destructor* to pre-adult bees during their development and can be found in all host body parts, including the brain^43^,^44^.

Here we ask if infection levels of these two fundamentally different pathogen types affect 1) behavioural performance i.e. flight characteristics and spatial exploration of the landscape, and 2) spatial exploration patterns in orientating bees, i.e. consistency with an optimized Lévy-flight searching pattern. *Nosema* is expected to have an adverse effect on flight performance due to its interference with bees’ metabolism^42^,^45^. Viruses, on the other hand, are not known to affect flight energetics but were found to alter a bee’s in-hive behaviour^46^. However, based on previous work on *temporal* fractals^21^,^24^,^25^, we hypothesize that high pathogen levels, particularly of viruses detected in the neural system, result in deficient search patterns of reduced fractal complexity, i.e. in a suppression of optimal Lévy flight characteristics. Our study provides the first insights into the effects of these physiological stressors on the robustness of navigational optimization strategies in animals.

## Material and Methods

**Figure 1:**
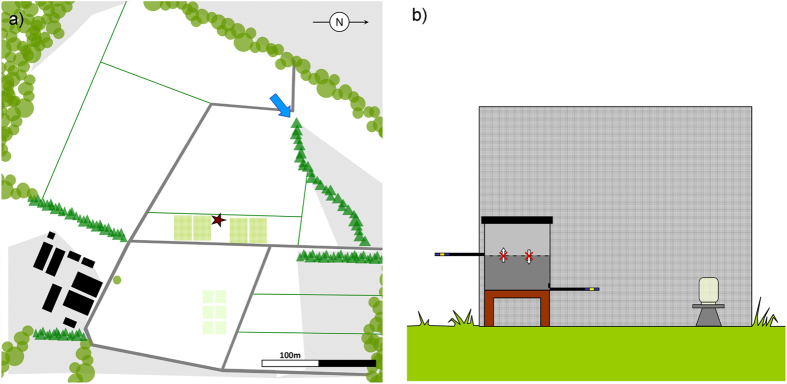
(**a**) Schematic map of the study area at Rothamsted Research (51°48′47.44′′, 0°22′45.74′′W) indicating the positions of the caged hive (red star) and the radar (blue arrow, Fig. S1b) in the agricultural landscape (white: non-flowering crop/harvested fields; green lines: field margins; light green areas close to the colony: pasture including flowering plants; pale green squares: maize plots potentially impairing tracking; dark grey: field tracks; green triangles: hedges; light green circles: trees/woodland; black squares: buildings). Areas with impaired radar tracking are shown in light grey. The distance between the radar and the colony is 235 m. [Map created by S Wolf, MS Powerpoint 2010.] (**b**) Caged colony setup consisting of a two-part honeybee hive divided by an odour-permeable mesh with bees inhabiting the lower brood box while not being able to access the upper brood box, a surrounding flight cage (3 m × 3 m × 1.8 m), a pollen feeder (not shown) and a gravity feeder for sucrose. The lower brood box allowed the bees to enter into the flight cage via a Perspex landing strip. The upper brood box contained a frame of comb and exited to the landscape around the cage via an identical landing strip. Bees transferred to the upper brood box or to the outgoing landing platform were used for tracking (Fig. S1a).

We tracked landscape-naïve honeybees on their first orientation flight after being released from a flight cage into an unknown landscape (Fig. 1) using a stationary, horizontally scanning radar^45^,^47^. A colony (host colony, HC) was placed in an insect-proof flight cage (3 m × 3 m × 1.8 m) located next to a grass field margin within flat and harvested farmland at Rothamsted Farm, UK, providing a suitable arena for radar tracking of bees over several hundred metres.

The hive consisted of two stacked brood boxes separated by a mesh, with the lower one containing the colony and opening into the flight cage via a Perspex tunnel, allowing the bees to forage on a sucrose-gravity feeder and a pollen feeder. The top brood box could not be entered by the bees and contained unpopulated honeycomb onto which individual foragers were placed prior to tracking. This ensured the test bees naturally embarked on foraging when leaving the hive via a similar Perspex tunnel (dummy exit) yet leading to the outside of the cage so that they could explore the novel landscape outside of the cage (Fig. 1).

All experimental bees were obtained from naturally *Varroa*-infected donor colonies (DCs) from Rothamsted Research stock. *Varroa* mite-fall over 10 days, a standard beekeeping practice to assess a colony’s *Varroa* infestation, served as a proxy indicator for putatively high and low virus-infection probability and virus load of the donor colonies^48^. Mature brood frames were taken from the DCs and were transferred into an incubator (34.5 °C) until the bees emerged. Two to three days after emergence the worker bees were marked individually with numbered queen marking tags (Opalithplättchen, Bienen-Voigt and Warnholz GmbH and Co. KG, Germany) and were randomly separated into two identical holding cages.

These groups of bees were subsequently bulk-fed either with a freshly prepared *Nosema*-inoculum (500 μl of 40% sucrose (w/w) containing 250,000 spores per bee) (NOS) or with 500 μl of 40% sucrose, free of *Nosema* spores but otherwise identical to the *Nosema*-inoculum (C)^45^ in order to ensure that *Nosema* infection was present in at least half of the bees. Six hours after inoculation, all bees were introduced to the HC, where they developed under identical natural conditions into foragers. Tracking bees from the same cohort allowed us to control for behavioural effects of age.

Marked foragers at least 14 days old and previously observed foraging at the feeder were collected from the hive entrance using a queen marking cage and transferred into the top brood box. Upon leaving the hive through the dummy exit they were briefly contained in the tunnel with shutters and equipped with a transponder before being released and allowed to exit the colony into the landscape. Only one bee was released and tracked at any time. Returning bees were caught when landing at the dummy entrance or on the external surface of the cage netting. These bees were subsequently frozen at −80 °C until pathogen screening. Released bees returning no radar signal for 45 min were declared lost. All experiments were conducted in favourable weather conditions (ambient temperature > 15 °C, no rain, no or light wind) from June to September 2012 and 2013.

The radar obtained positional fixes (range (m) and bearing (rad)) every 3 s (20 rpm rotational scan)^45^ (see Supplementary Information, Fig. S2) from which we reconstructed the bees’ flight trajectories and inferred flight speed, total stop time, total track length, and maximal displacement distance (furthest location of the bee from the colony) as measures of flight performance. Based on the smallest polygonal hull completely enclosing the flight track we compared the spatial characteristics of the orientation flights using area and perimeter of the hull and isoperimetry; i.e. circularity of the track hull (Eq. S1 in Supplementary Information).

It is not possible to precisely control the ontogeny of infection of DWV or *Nosema* in individual bees, where infection success and recurring horizontal transmission may lead to high variation in actual pathogen loads. To account for this, all tracked bees were analysed post-hoc for their disease load at the time of their orientation flight, and these pathogen loads were used for all statistical analyses. Post-hoc virus screening of tracked bees was done individually via qRT-PCR (primers: DWV: DWV-F2, DWV-R2a; VDV: VDV-F2, VDV-R2a^49^) based on cDNA (reverse-transcribed RNA) obtained from the bees’ head, using RP49 as housekeeping gene. A negative control containing RNA-free HPLC-water, and a virus-positive abdominal cDNA sample were included as controls in each reaction run. We used an upper Ct – value of 35 cycles to minimize the risk of false positives^50^,^51^. Using a DWV standard curve for qPCR efficiency, we inferred virus loads per head of a bee. *Nosema* spore loads were assessed microscopically from dissected mid-guts using a haemocytometer following standard protocol^52^,^53^. Based on these diagnostics, the tracked bees were assigned to the following groups: *Nosema*-free (representing a *Nosema* control group), low *Nosema* infection (<1000 spores/μl) and high infection (>1000 spores/μl), as well as presence (Ct-value < 35) and absence (Ct-value > 35) of DWV (Table 1). These form the basis of robust comparative statistics of our highly variable behavioural and pathological data-set to unravel the pathogen effects on flight performance (Tables 1 and 2, Supplementary Fig. S3).

**Figure 2:**
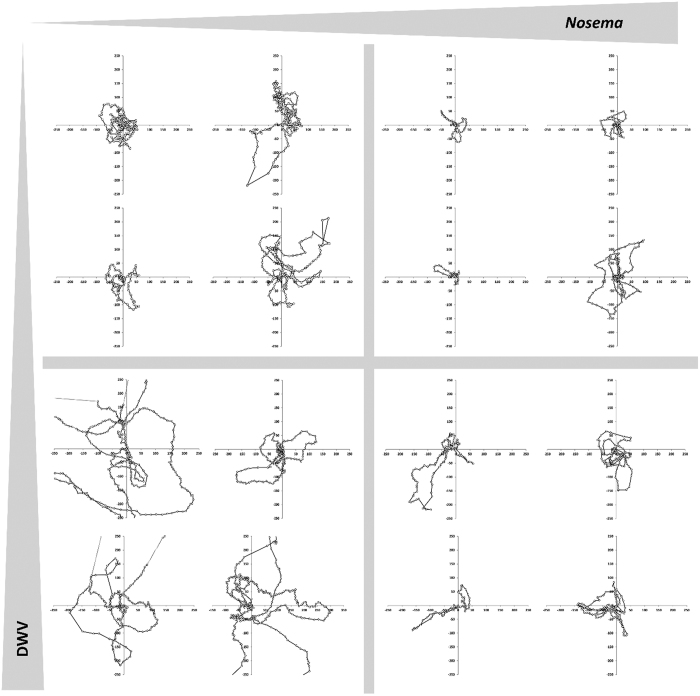
Representative orientation flight tracks showing the movements of bees with increasing *Nosema* infection levels (left and right column, respectively) and infection with DWV (absence/presence; upper and lower row, respectively). Each plot shows the bees’ flight path within a 25 ha coordinate system, with the colony at the origin and geographic orientation corresponding to the map of study area in Fig. 1. The position of the bee (circles) is recorded every 3 seconds (see main text and Supplementary Information for details). Discontinuously recorded flight trajectories are given as a dotted line. There is a significant negative effect of *Nosema* infection on track spatial scale. Virus infection corresponded to spatially expanded tracks, albeit this trend was non-significant. [Created by S Wolf, MS Excel 2010].

**Figure 3:**
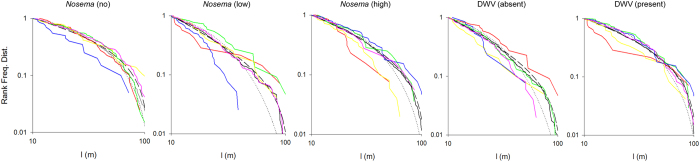
Frequency rank distributions (FRDs) of flight step lengths (l in metres) for all bees in each pathogen group (black solid lines) together with the group-level best-fit power-law distributions (black dashed-line) and the group-level best fit exponential distributions (black dotted lines). For each pathogen group the FRDs are better represented by power-laws (see also Fig. S4), which are indicative of Lévy flight patterns. For *pooled* flight data per pathogen group (black solid line), the fit to power-laws (black dashed line) is highly supported (Akaike weights AIC = 1.00). In each group > 50% of the *individual* tracks have a maximum likelihood estimate for the power-law (Lévy) exponent of μ ≈ 2.1 supported by Akaike weights for a power-law of AIC >0.5. Some examples of FRD for individual bees are shown for comparison (coloured lines).

We employed the same five pathogen groups of bees (3 *Nosema* groups and 2 DWV groups) for a robust and reliable comparison of Lévy flight characteristics. We focussed on both *pooled* flight data per pathogen-group as well as *individual* flight trajectories in these groups to compare Lévy exponents and the Akaike weights for power-laws in the context of pathogen load. (Tables 2 and 3 and Figs 2, 3 and S4). In both approaches we tested for the presence of optimal Lévy flight patterns following the approach of Reynolds *et al.*^18^. The analysis is based on flight path-derived sequences of straight-line movements between points (turning points) of significant directional change. These turning points are defined by a directional change between the flight direction immediately before and after the putative turning point of more than 90°. Statistical properties of these path representations do not change significantly when the critical angle of 90° is changed by ±30°. Following well-established practice^54^, flight-length distributions were then fitted to power-law distributions (indicative of Lévy flights) and to exponential distributions (a null distribution) using maximum likelihood methods^55^. These model distributions are prescribed by


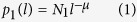



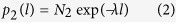


where 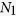 and 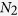 are normalization factors which ensure that the frequency distributions sum correctly to unity when integrated over all flight-lengths between the lower and upper cut-offs, μ is the power-law exponent (also called the Lévy exponent) and λ is the decay rate of the exponential. The lower cut-off was taken to be 10 m (which is comparable to the shortest step that can be resolved by the radar), and the upper cut-off was taken to be length of the longest flight segment in the dataset under analysis. The best-fitting model distribution was identified using the Akaike information criterion, the weight of which ranges from 0 (no support for the model) to 1 (full support for the model).

Both flight parameters and individual Lévy exponents (Table 3) for each of the pathogen groups were compared using a linear mixed model (LMM) with restricted maximum likelihood (REML) and a fully crossed fixed model (*Nosema* infection level (no/low/high) × virus-infection (presence/absence)), with tracking day as a random factor. Pathogen interactions were tested excluding individuals free of *Nosema* (n = 16). In the absence of significant interactions, the interaction-term was dropped from the LMM for statistical comparison of the pathogen effects on the flight parameters and Lévy exponents (Table 2).

All statistical analyses were performed using the statistical software GenStat V17.1. (VSN International, 2011).

## Results

### Flight performance

In total, 78 bees were successfully tracked on their first orientation flights and successfully pathogen-screened. The average *Nosema* infection level was 4.79 × 10^3^ spores/μl bee gut extract (range: 0–2.36 × 10^4^) (equivalent to an extrapolated mean of 2.4 × 10^6 ^spores per bee) and they were naturally infected with an average of 1.42 × 10^10^ copies of DWV per bee head (range: 0–1.99 × 10^11^) (Supplementary Fig. S2). 45% and 26% of all bees were single-infected with DWV or *Nosema*, respectively, with the rest of the bees showing co-infection (Table 1).

We found that the spatial scale of the orientation flight was significantly reduced in bees with *Nosema* infection in comparison to bees free of spores (Table 2; Fig. 2, Supplementary Fig. S3) with infection intensity playing a secondary role in shaping behavioural performance. While orientation flights of bees with low and high *Nosema* loads covered on average an area of 34,353 m^2^ and 20,390 m^2^, respectively, the orientation flights were over three times larger for bees with no *Nosema* infection (127,123 m^2^). Similarly, we found a significant *Nosema*-associated reduction in overall track length and track perimeter, both of which dropped by half with *Nosema* infection, but differed only marginally between high and low infection levels (Table 2, Supplementary Fig. S3); the maximum displacement distance only reached an average of 148 m in highly infected bees compared to 317 m for bees with no *Nosema* infection.

While total flight time was significantly longer in *Nosema*-clean bees, we also find a surprising and significant increase in total stop time in *Nosema*-clean bees, which is typically associated with *Nosema* infection. We attribute this to the overall longer activity time of these *Nosema*-clean bees, allowing or indeed requiring longer rest times (Table 2). Testing the track area the bees covered during the bees’ actual flight time (excluding stop time), we find that *Nosema* infected bees explore >65% less area per time unit flown than *Nosema*-free bees.

In contrast, none of the measured parameters varied significantly with the presence or absence of DWV nor was there any significant interaction between the two pathogen types for any parameter (Table 2). In response, we removed the non-significant interaction-term from the statistical comparison of pathogen effects. In no case did this approach affect the results of our initial analysis. Though not significant, there is an interesting tendency for larger orientation flights in DWV-infected bees that warrants further investigation (Table 2, Fig. 2).

In line with previous work^45^, the mean flight speed was affected by neither *Nosema* nor Deformed wing virus. Likewise, the circularity (isoperimetry) of orientation flights was not significantly affected by either pathogen (Table 2).

### Lévy flights

Analysing the *individual* bee movement patterns in relation to their disease load, we found no significant difference in the prevalence of Lévy flight characteristics (AIC >0.5) between bees infected with either *Nosema* or DWV as compared to bees without these pathogens (among *Nosema* categories: *χ*^*2*^ = 0.06, *d.f.* = 2, *p* = 0.97; between DWV categories: *χ*^*2*^ = 0.22, *d.f.* = 1, *p* = 0.64) (Fig. 3, Table 3). Overall 54% of the bee tracks indicated Lévy exponents of μ ≈ 2 (Tables 2 and 3), which were supported by Akaike weights > 0.5.

In addition, and in contrast to our hypothesis, we find no evidence for reduced fractal complexity i.e. lower Lévy exponents, as a function of pathogen infection. Comparing all Lévy exponents of *individual* tracks (Fig. 3), which were supported by individual AIC’s of >0.5, we find that *Nosema*-free bees exhibit a mean Lévy exponent of μ = 2.17, whereas bees with low or high *Nosema* spore load showed mean Lévy exponents of μ = 2.27 and μ = 2.24 (standard error of difference (s.e.d.) = 0.16), respectively. There was no significant difference in the Lévy exponents with respect to *Nosema* infection (*p* = 0.86, Table 2). Similarly, μ was 2.28 and 2.17 (s.e.d. = 0.14) for bees without and with Deformed wing virus, respectively, and did not differ significantly (*p* = 0.62, Table 2).

Based on data *pooled* across all bees in a specific pathogen group the Akaike weights for power-laws (mean μ_POOLED_ ≈ 2.1) were 1.00, thus giving full support for Lévy-flights in each of the pathogen groups as is clearly illustrated in Figs 3 and S4. Our data indicate that the two focal pathogens do not lead to a significant reduction in fractal complexity in the spatio-temporal domain, contrasting with the pathogen-sensitive temporal fractals (Figs 3 and S4, Table 2).

Though warranting caution in the interpretation, there is an interesting tendency for the Lévy exponent to slightly increase with *Nosema* load, i.e., for searching to become sub-optimal and closer to being scale-finite rather than scale-free (as μ > 3 corresponds to Brownian (scale-finite) flight patterns). However, since *Nosema* infection may reduce the size of the search flights, longer flight segments may be curtailed in these cases. Such truncations (under-representation) of longer flight segments will tend to increase the maximum likelihood estimates for μ, effectively over-estimating the exponent without biological causation. The reverse may apply for DWV infection where the presence of DWV, loosely associated with spatially more expansive tracks, seems to lead to a Lévy exponent closer to 2 (μ = 2.17), very similar to the exponents found for *Nosema*-free bees. It remains to be investigated if these (non-significant) variations in Lévy exponents indeed foreshadow subtle, yet genuine effects of pathogen infections on orientation flight characteristics and are thus biologically relevant.

Overall, our data indicate that Lévy flights are not only a common feature of honeybee orientation flights, being the most parsimonious flight-pattern model in 54% of the colony workforce of foragers, but also that they are robust against otherwise marring effects of pathogen infection

## Discussion

Fractal patterns are a widespread and robust feature in animal behaviour^27^,^56^,^57^,^58^ and have been implicated in facilitating an animal’s ability to efficiently respond to environmental change^5^,^56^,^59^,^60^. However, while the underlying principles of time allocation to given behaviours have been intensely studied in the context of the internal state of an animal, investigations in the spatial domain are thus far restricted to environmental characteristics, a fact that has been highlighted as a substantial shortcoming to our understanding of the occurrence, origin and general characteristics of scale-free patterns in animal behaviour^20^,^27^.

Using honeybee orientation flights as a model to investigate if and how pathogens may affect spatial search patterns, we show for the first time that, while the *size and duration* of the orientation flight was clearly associated with *Nosema* load, the underlying *search algorithm* was not. During their orientation flights, honeybees and bumblebees “collect” salient visual cues such as prominent landmarks (trees, houses, etc.) and linear landscape features (roads, hedges, etc.)^34^ in spatially increasing search flights^36^ that aid their successful navigation between the colony and foraging grounds^35^. Efficient acquisition of such information is thus a pre-requisite for successful foraging, and any impairment of this ability can be assumed to negatively affect individual and colony performance. In line with previous studies on search flights of honeybees^16^,^17^,^18^,^38^ and orienting bumblebees^37^, we find that our orientation flight data not only followed the expected optimal Lévy distribution, a hallmark of highly efficient environmental exploration, but that these *search patterns* were robust against both *Nosema* and virus infection, with disease load having no significant effect on either the Lévy prevalence or the numerical value of the Lévy exponent.

In contrast, for the *flight performance* we find that even bees with moderate *Nosema* infection levels, likely to be commonly found in naturally infected bees^61^, exhibit significant impairments. Infected bees covered on average only a third of the area during their orientation flights and flew a shorter distance from the hive (a reduction of over 41% in maximum displacement distance) in comparison to bees which had no *Nosema* infection. This is in line with previous studies showing that *Nosema* sp., an obligatory gut pathogen infecting intestinal cells, interferes with honeybee energetics^42^,^62^ and affects flight performance^45^,^63^,^64^. For infections with DWV, we see putatively infection-induced changes in some track parameters, albeit non-significant; as DWV may alter in-hive behaviour of honeybees^46^, such changes in flight performance warrant further investigation. Other flight parameters such as a bee’s mean flight speed (∅_total_ = 3.42 ms^−1^ ± 0.58 ms^−1^) closely matches previously reported estimates of honeybee flight speed (3.6 ms^−1^, range: 0.6 ms^−1^–6.2 ms^−1^ 36; 3.19 ms^−1^, range: 2.86 ms^−1^–3.53 ms^−1^ 45) and did not vary significantly with pathogen load.

It remains to be studied if and how the *Nosema*-induced spatial reduction of a honeybee’s orientation flights translate into deficits in foraging performance, or even prevents bees becoming successful foragers reliably able to navigate through the wider landscape. The reduced access to landscape features is unlikely to be compensated by an efficient search strategy, suggesting that these effects may play an important yet cryptic and thus far unreported role in poor colony fitness observed in various regions of the world^40^,^65^.

While the effects on bee flight performance are in line with previous reports, the robustness of the fractal characteristics of the orientation flight (optimal Lévy flight, μ ≈ 2) is not. Our findings are in contrast to temporal fractal patterns governing the time-allocation to behavioural routines, which was shown to be highly sensitive to diseases and parasites, among other stressors, leading to more stereotypical and less optimal behaviours in infected as compared to healthy individuals^21^,^24^,^25^,^26^.

This pronounced contrast in robustness of temporal versus spatial scale-free patterns raises interesting questions about the origin of fractal features in animal behaviour^27^,^57^. The fact that pathogens, though clearly affecting the bees’ flight behaviour, do not interfere with the search patterns suggests that Lévy flights are not a sophisticated cognitive skill, which can be assumed to be costly to acquire and to maintain; and would be potentially prone to interference from stressors. Rather, such fundamental characteristics in animal movement may result from innate neuronal stochasticity and accumulated sensory imprecision of the decision making process^27^,^66^,^67^, similar to the universal activity patterns found in cue-deprived insects^58^.

Our data support the idea that Lévy flight characteristics may not be the product of direct selection but that, conversely, selection favours individuals *not losing* their innate ability to optimally Lévy-fly^27^,^57^. In fact, MacIntosh^27^ highlighted ‘error tolerance’, i.e. the ability to maintain Lévy-like movement patterns under adverse conditions such as pathogen infection, as a potentially strong selective force that may explain the widespread occurrence of Lévy flights^27^. We provide an interesting new insight into the evolutionary foundations of optimal movement patterns in animals, which we hope will inspire further research in this little explored field.

## Additional Information

**How to cite this article**: Wolf, S. *et al.* Optimal search patterns in honeybee orientation flights are robust against emerging infectious diseases. *Sci. Rep.* **6**, 32612; doi: 10.1038/srep32612 (2016).

## Supplementary Material

Supplementary Information

## Figures and Tables

**Table 1:** Sample sizes of tracked bees for each pathogen load category.

	no *Nosema* (0 spores/μl)	low *Nosema* (<1000 spores/μl)	high *Nosema* (>1000 spores/μl)	Total
DWV absent	0	7	20	27
DWV present	16	19	16	51
Total	16	26	36	78

**Table 2:** Mean track parameters and *individual* Lévy exponents (predicted means and standard error of difference, s.e.d.) in the context of infection with *Nosema* sp. and DWV.

	*Nosema* sp.					DWV				*Nosema* × DWV
	no (0 spores/μl)	low (<1000 spores/μl)	high (>1000 spores/μl)	s.e.d.	LMM (REML)	absence	presence	s.e.d.	LMM (REML)	LMM (REML)
	mean (n = 16)	mean (n = 26)	mean (n = 36)			mean (n = 27)	mean (n = 51)			
track area	126,123 m^2^	34,353 m^2^	20,390 m^2^	19,640 m^2^	*F*^NOS^_2, 44.4_ = 17.8, ***p***** < 0.001**	59.372 m^2^	61,205 m^2^	17,645 m^2^	*F*^DWV^_1, 73.1_ = 0.01, *p* = 0.92	*F*^NOS*DWV^_1, 58.0_ = 0.58, *p* = 0.45
track perimeter	1585 m	834 m	636 m	197.2 m	*F*^NOS^_2, 49.9_ = 11.1, ***p***** < 0.001**	682 m	918 m	251 3 m	*F*^DWV^_1, 73.1_ = 0.08, *p* = 0.78	*F*^NOS*DWV^_1, 57.9_ = 0.18, *p* = 0.68
isoperimetry	0.51	0.42	0.43	0.04	*F*^NOS^_2, 54.9_ = 2.73, *p* = 0.08	0.45	0.46	0.03	*F*^DWV^_1, 73.1_ = 0.00, *p* = 0.95	*F*^NOS*DWV^_1, 57.9_ = 0.75, *p* = 0.39
max. displacement distance	317 m	186 m	148 m	41.3 m	*F*^NOS^_2, 50.5_ = 10.5, ***p***** < 0.001**	213 m	222 m	36.9 m	*F*^DWV^_1, 73.8_ = 0.06, *p* = 0.81	*F*^NOS*DWV^_1, 57.9_ = 0.48, *p* = 0.49
total track length	3795 m	1793 m	1863 m	697.0 m	*F*^NOS^_2, 55.5_ = 4.74, ***p***** = 0.013**	1843 m	2354 m	602.3 m	*F*^DWV^_1, 72.7_ = 0.03, *p* = 0.87	*F*^NOS*DWV^_1, 57.0_ = 1.79, *p* = 0.19
total flight time	925 s	817 s	520 s	211.0 s	*F*^NOS^_2, 62.1_ = 2.75, *p* = 0.071	511 s	757 s	170.3 s	*F*^DWV^_1, 72.6_ = 1.01, *p* = 0.32	*F*^NOS*DWV^_1, 57.9_ = 2.74, *p* = 0.10
total stop time	915 s	462 s	468 s	159.1 s	*F*^NOS^_2, 52.3_ = 4.43, ***p***** = 0.017**	602 s	628 s	145.4 s	*F*^DWV^_1, 73.1_ = 0.03, *p* = 0.85	*F*^NOS*DWV^_1, 57.8_ = 1.36, *p* = 0.25
flight speed	3.63 ms^−1^	3.32 ms^−1^	3.37 ms^−1^	0.19 ms^−1^	*F*^NOS^_2, 51.8_ = 1.56, *p* = 0.22	3.41 ms^−1^	3.47 ms^−1^	0.16 ms^−1^	*F*^DWV^_1, 72.2_ = 0.14, *p* = 0.71	*F*^NOS*DWV^_1, 57.0_ = 0.39, *p* = 0.53
area : flight time	289.9 m^2^s^−1^	69.7 m^2^s^−1^	39.9 m^2^s^−1^	58.83 m^2^s^−1^	*F*^NOS^_2, 42.2_ = 10.19, ***p***** < 0.001**	153.9 m^2^s^−1^	112.4 m^2^s^−1^	55.6 m^2^s^−1^	*F*^DWV^_1, 74.0_ = 0.56, *p* = 0.46	*F*^NOS*DWV^_1, 57.9_ = 1.91, *p* = 0.17
Lévy exponent *μ* (total)	2.15	2.31	2.25	0.11	*F*^NOS^_2, 31.9_ = 0.98, *p* = 0.39	2.37	2.11	0.10	*F*^DWV^_1, 51.9_ = 7.01, ***p***** = 0.01**	*F*^NOS*DWV^_1, 51.0_ = 0.56, *p* = 0.46
Lévy exponent *μ* (AIC > 0.5)	2.17	2.27	2.24	0.16	*F*^NOS^_2, 21.8_ = 0.16, *p* = 0.86	2.28	2.17	0.14	*F*^DWV^_1, 26.7_ = 0.50, *p* = 0.48	*F*^NOS*DWV^_1, 25.6_ = 0.25, *p* = 0.62

Parameters were calculated from the radar track data and were statistically compared using a Linear Mixed Model (LMM) with Restricted Maximum Likelihood (REML). Lévy exponents were compared for both the complete dataset (ignoring Akaike weights) and for those supported by Akaike weights >0.5. The *Nosema* × DWV interaction included only bees with detected *Nosema* infection (n = 62) excluding *Nosema*-free individuals (n = 16). In the absence of significant interactions, we dropped the interaction-term from the LMM (i.e. main effects only) for more robust comparisons of pathogen effects.

**Table 3:** List of results from the Levy flight analysis of the *individual* bee tracks in each of the five pathogen groups.

Bee ID	# flight segments	Akaike weight	*μ*		Bee ID	# flight segments	Akaike weight	*μ*
**no** ***Nosema***					**no DWV**			
180812a_Gr	43	0.86	***1.93***		020913a_R	39	0.09	2.87
180812d_Wh	121	0.95	***1.89***		020913h_Bl2	30	0.32	2.56
190812c_R	26	0.01	1.75		040913e_R	38	0.96	***2.18***
190812f_Gr	94	0.27	1.91		040913h_Gr2	50	0.13	2.41
190812h_Gr	29	0.44	2.03		040913l_Gr2	39	0.69	***1.99***
220812e_Wh	31	0.85	***2.01***		140813a_Y	29	0.54	***2.03***
220812f_Gr	21	0.65	***2.17***		140813c_Y	21	0.87	***2.82***
230812a_Wh	31	0.09	2.13		140813d_Y	13	0.2	2.77
230812c_R	20	0.73	***2.18***		190713b_Bl1	61	0.57	***1.9***
230812e_Wh	34	0.55	***2.53***		190813a_Y	36	0.19	2.67
260812c_Wh	89	0.25	1.92		210813e_Y	94	0.67	***2.08***
260812j_R	127	0.01	1.86		260813g_Gr2	22	0.59	***2.05***
**low** ***Nosema***					280813g_Wh	13	0.21	2.56
020913a_R	39	0.09	2.87		280813h_R	107	0.67	***2.26***
140813c_Y	21	0.87	***2.82***		290813d_Bl2	16	0.98	***3.25***
150912c_Gr	66	0.48	1.93		290813n_Bl2	21	0.35	2.41
150912e_Wh	13	0.36	2.54		290813o_Gr2	18	0.62	***2.18***
180812f_Wh	10	0.6	2.55		290813q_Gr2	28	0.12	2.16
180812i_R	36	0.89	***1.92***		290813r_Bl2	7	0.1	1.73
190812a_Wh	9	0.18	2.11		310813g_Bl2	11	0.44	2.53
190812b_Wh	39	0.54	***2.09***		**DWV**			
220812a_R	17	0.42	2.14		020913j_Gr2	28	0.54	***2.06***
220812b_Gr	76	0.92	***2.12***		150713a_Bl1	183	1	***1.93***
230812l_R	43	0.8	***1.9***		150912c_Gr	66	0.48	1.93
260812a_R	53	0.12	2.16		150912e_Wh	13	0.36	2.54
260812i_Gr	41	0.2	2.1		180812a_Gr	43	0.86	***1.93***
260813g_Gr2	22	0.59	***2.05***		180812d_Wh	121	0.95	***1.89***
270813a_Gr2	34	0.36	1.95		180812f_Wh	10	0.6	2.55
280812c_Wh	20	0.95	***2.35***		180812i_R	36	0.89	***1.92***
280813e_Wh	14	0.99	***2.59***		190713a_Gr1	121	0.44	2
280813h_R	107	0.67	***2.26***		190713c_Gr1	43	0.67	***1.97***
290813f_Gr2	34	0.72	***2***		190812a_Wh	9	0.18	2.11
**high** ***Nosema***					190812b_Wh	39	0.54	***2.09***
020913h_Bl2	30	0.32	2.56		190812c_R	26	0.01	1.75
020913j_Gr2	28	0.54	***2.06***		190812f_Gr	94	0.27	1.91
040913e_R	38	0.96	***2.18***		190812h_Gr	29	0.44	2.03
040913h_Gr2	50	0.13	2.41		220812a_R	17	0.42	2.14
040913l_Gr2	39	0.69	***1.99***		220812b_Gr	76	0.92	***2.12***
140813a_Y	29	0.54	***2.03***		220812e_Wh	31	0.85	***2.01***
140813d_Y	13	0.2	2.77		220812f_Gr	21	0.65	***2.17***
150713a_Bl1	183	1	***1.93***		230812a_Wh	31	0.09	2.13
190713a_Gr1	121	0.44	2		230812c_R	20	0.73	***2.18***
190713b_Bl1	61	0.57	***1.9***		230812e_Wh	34	0.55	***2.53***
190713c_Gr1	43	0.67	***1.97***		230812l_R	43	0.8	***1.9***
190813a_Y	36	0.19	2.67		260812a_R	53	0.12	2.16
270813i_Gr2	5	0.17	1.83		260812c_Wh	89	0.25	1.92
280813g_Wh	13	0.21	2.56		260812i_Gr	41	0.2	2.1
290813b_Bl2	38	0.56	***1.99***		260812j_R	127	0.01	1.86
290813d_Bl2	16	0.98	***3.25***		270813a_Gr2	34	0.36	1.95
290813g_Bl2	17	0.58	***2.46***		270813i_Gr2	5	0.17	1.83
290813j_Bl2	16	0.62	***2.39***		280812c_Wh	20	0.95	***2.35***
290813n_Bl2	21	0.35	2.41		280813e_Wh	14	0.99	***2.59***
290813o_Gr2	18	0.62	***2.18***		290813b_Bl2	38	0.56	***1.99***
290813q_Gr2	28	0.12	2.16		290813f_Gr2	34	0.72	***2***
290813r_Bl2	7	0.1	1.73		290813g_Bl2	17	0.58	***2.46***
300813b_Bl2	16	0.72	***2.05***		290813j_Bl2	16	0.62	***2.39***
310813g_Bl2	11	0.44	2.53		300813b_Bl2	16	0.72	***2.05***

Flight segments denote the number of linear flight steps between distinct turning points (see methods). Individual estimates of the power-law exponent μ (indicative of a Lévy flight when μ ≈ 2) supported by Akaike weights >0.5 are given in bold italics.

## References

[b1] Dall S. R., Giraldeau L. A., Olsson O., McNamara J. M. & Stephens D. W. Information and its use by animals in evolutionary ecology. Trends Ecol. Evol. 20, 187–193, doi: 10.1016/j.tree.2005.01.010 (2005). 16701367

[b2] Bell W. J. Searching behavior patterns in insects. Annual Review of Entomology 35, 447–467 (1990).

[b3] Viswanathan G. *et al.* Lévy flight search patterns of wandering albatrosses. Nature 381, 413–415 (1996). 10.1038/nature06199 17960243

[b4] Bartumeus F. & Catalan J. Optimal search behavior and classic foraging theory. Journal of Physics A: Mathematical and Theoretical 42, 434002 (2009).

[b5] Bartumeus F., Catalan J., Fulco U., Lyra M. & Viswanathan G. Optimizing the encounter rate in biological interactions: Lévy versus Brownian strategies. Physical Review Letters 88, 097901 (2002). 11864054 10.1103/PhysRevLett.88.097901

[b6] Reynolds A. M. & Rhodes C. J. The Lévy flight paradigm: random search patterns and mechanisms. Ecology 90, 877–887, doi: 10.1890/08-0153.1 (2009). 19449680

[b7] Bartumeus F., Peters F., Pueyo S., Marrasé C. & Catalan J. Helical Lévy walks: adjusting searching statistics to resource availability in microzooplankton. Proceedings of the National Academy of Sciences 100, 12771–12775 (2003). 10.1073/pnas.2137243100 PMC240693 14566048

[b8] Sims D. W. *et al.* Hierarchical random walks in trace fossils and the origin of optimal search behavior. Proceedings of the National Academy of Sciences 111, 11073–11078 (2014). 10.1073/pnas.1405966111 PMC4121825 25024221

[b9] Sims D. W. *et al.* Scaling laws of marine predator search behaviour. Nature 451, 1098–1102, doi: http://www.nature.com/nature/journal/v451/n7182/suppinfo/nature06518_S1.html (2008). 18305542 10.1038/nature06518

[b10] Humphries N. E. *et al.* Environmental context explains Lévy and Brownian movement patterns of marine predators. Nature 465, 1066–1069 (2010). 20531470 10.1038/nature09116

[b11] Hays G. C. *et al.* High activity and Lévy searches: jellyfish can search the water column like fish. Proceedings of the Royal Society B: Biological Sciences 279, 465–473 (2012). 21752825 10.1098/rspb.2011.0978 PMC3234559

[b12] Korobkova E., Emonet T., Vilar J. M., Shimizu T. S. & Cluzel P. From molecular noise to behavioural variability in a single bacterium. Nature 428, 574–578 (2004). 15058306 10.1038/nature02404

[b13] Harris T. H. *et al.* Generalized Lévy walks and the role of chemokines in migration of effector CD8+T cells. Nature 486, 545–548 (2012). 22722867 10.1038/nature11098 PMC3387349

[b14] Raichlen D. A. *et al.* Evidence of Lévy walk foraging patterns in human hunter–gatherers. Proceedings of the National Academy of Sciences 111, 728–733 (2014). 10.1073/pnas.1318616111 PMC3896191 24367098

[b15] Reynolds A. M. Fitness-maximizing foragers can use information about patch quality to decide how to search for and within patches: optimal Lévy walk searching patterns from optimal foraging theory. J. R. Soc. Interface 9, 1568–1575, doi: 10.1098/rsif.2011.0815 (2012). 22258553 PMC3367820

[b16] Reynolds A. M., Smith A. D., Reynolds D. R., Carreck N. L. & Osborne J. L. Honeybees perform optimal scale-free searching flights when attempting to locate a food source. Journal of Experimental Biology 210, 3763–3770, doi: 10.1242/jeb.009563 (2007). 17951417

[b17] Reynolds A. M., Swain J. L., Smith A. D., Martin A. P. & Osborne J. L. Honeybees use a Lévy flight search strategy and odour-mediated anemotaxis to relocate food sources. Behavioral Ecology and Sociobiology 64, 115–123, doi: 10.1007/s00265-009-0826-2 (2009).

[b18] Reynolds A. M. *et al.* Displaced honey bees perform optimal scale-free search flights. Ecology 88, 1955–1961, doi: 10.1890/06-1916.1 (2007). 17824426

[b19] Humphries N. E., Weimerskirch H., Queiroz N., Southall E. J. & Sims D. W. Foraging success of biological Lévy flights recorded *in situ*. Proceedings of the National Academy of Sciences 109, 7169–7174 (2012). 10.1073/pnas.1121201109 PMC3358854 22529349

[b20] MacIntosh A. J., Pelletier L., Chiaradia A., Kato A. & Ropert-Coudert Y. Temporal fractals in seabird foraging behaviour: diving through the scales of time. Scientific Reports 3 (2013). 10.1038/srep01884 PMC3662970 23703258

[b21] MacIntosh A. J., Alados C. L. & Huffman M. A. Fractal analysis of behaviour in a wild primate: behavioural complexity in health and disease. J. R. Soc. Interface 8, 1497–1509 (2011). 21429908 10.1098/rsif.2011.0049 PMC3163426

[b22] Shimada I., Minesaki Y. & Hara H. Temporal fractal in the feeding behavior of *Drosophila melanogaster*. J. Ethol. 13, 153–158 (1995).

[b23] Kembro J. M., Perillo M. A., Pury P. A., Satterlee D. G. & Marin R. H. Fractal analysis of the ambulation pattern of Japanese quail. Br Poult Sci. 50, 161–170, doi: 10.1080/00071660802710116 (2009). 19373715

[b24] Alados C. L., Escos J. M. & Emlen J. M. Fractal structure of sequential behaviour patterns: an indicator of stress. Anim Behav. 51, 437–443 (1996).

[b25] Escós J. M., Alados C. L. & Emlen J. M. Fractal Structures and Fractal Functions as Disease Indicators. Oikos 74, 310–314, doi: 10.2307/3545661 (1995).

[b26] Seuront L. & Cribb N. Fractal analysis reveals pernicious stress levels related to boat presence and type in the indo–pacific bottlenose dolphin, Tursiops aduncus. Physica A 390, 2333–2339 (2011).

[b27] MacIntosh A. J. At the edge of chaos–error tolerance and the maintenance of Lévy statistics in animal movement Comment on “Liberating Lévy walk research from the shackles of optimal foraging” by AM Reynolds. Physics of Life Reviews 14, 105–107 (2015). 26205677 10.1016/j.plrev.2015.07.010

[b28] Winston M. L. The biology of the honeybee. (Harvard University Press, 1991).

[b29] Beekman M. & Ratnieks F. Long‐range foraging by the honeybee, *Apis mellifera* L. Functional Ecology 14, 490–496 (2000).

[b30] Cheung A. *et al.* Still no convincing evidence for cognitive map use by honeybees. Proceedings of the National Academy of Sciences 111, E4396–E4397 (2014). 10.1073/pnas.1413581111 PMC4210289 25277972

[b31] Wehner R. & Menzel R. Do insects have cognitive maps? Annual Review of Neuroscience 13, 403–414 (1990). 10.1146/annurev.ne.13.030190.002155 2183682

[b32] Menzel R. *et al.* Honey bees navigate according to a map-like spatial memory. Proceedings of the National Academy of Sciences of the United States of America 102, 3040–3045 (2005). 15710880 10.1073/pnas.0408550102 PMC549458

[b33] Collett T. S. & Collett M. Memory use in insect visual navigation. Nature Reviews Neuroscience 3, 542–552 (2002). 12094210 10.1038/nrn872

[b34] Collett T. S. & Graham P. Insect navigation: do honeybees learn to follow highways? Current Biology 25, R240–R242 (2015). 25784046 10.1016/j.cub.2014.11.003

[b35] Capaldi E. A. & Dyer F. C. The role of orientation flights on homing performance in honeybees. Journal of Experimental Biology 202, 1655–1666 (1999). 10333511 10.1242/jeb.202.12.1655

[b36] Capaldi E. A. *et al.* Ontogeny of orientation flight in the honeybee revealed by harmonic radar. Nature 403, 537–540 (2000). 10676960 10.1038/35000564

[b37] Osborne J. L. *et al.* The ontogeny of bumblebee flight trajectories: From naive explorers to experienced foragers. Plos one 8, doi: e78681.10.1371/journal.pone.0078681 (2013). 10.1371/journal.pone.0078681 PMC3827042 24265707

[b38] Reynolds A. M. Cooperative random Lévy flight searches and the flight patterns of honeybees. Phys. Lett. A 354, 384–388, doi: 10.1016/j.physleta.2006.01.086 (2006).

[b39] Ratnieks F. L. & Carreck N. L. Clarity on honey bee collapse? Science 327, 152–153 (2010). 20056879 10.1126/science.1185563

[b40] Potts S. G. *et al.* Global pollinator declines: trends, impacts and drivers. Trends Ecol. Evol. 25, 345–353 (2010). 20188434 10.1016/j.tree.2010.01.007

[b41] Fries I. *Nosema ceranae* in European honeybees (*Apis mellifera*). Journal of Invertebrate Pathology 103 Suppl 1, S73–S79, doi: 10.1016/j.jip.2009.06.017 (2010). 19909977

[b42] Mayack C. & Naug D. Energetic stress in the honeybee *Apis mellifera* from *Nosema ceranae* infection. Journal of Invertebrate Pathology 100, 185–188, doi: 10.1016/j.jip.2008.12.001 (2009). 19135448

[b43] de Miranda J. R. & Genersch E. Deformed Wing Virus. Journal of Invertebrate Pathology 103, S48–S61 (2010). 19909976 10.1016/j.jip.2009.06.012

[b44] Martin S. J. *et al.* Global honey bee viral landscape altered by a parasitic mite. Science 336, 1304–1306 (2012). 22679096 10.1126/science.1220941

[b45] Wolf S. *et al.* So near and yet so far: harmonic radar reveals reduced homing ability of Nosema infected honeybees. Plos one 9, e103989, doi: 10.1371/journal.pone.0103989 (2014). 25098331 PMC4123971

[b46] Natsopoulou M. E., McMahon D. P. & Paxton R. J. Parasites modulate within-colony activity and accelerate the temporal polyethism schedule of a social insect, the honeybee. Behavioral Ecology and Sociobiology 1–13 (2015). 10.1007/s00265-015-2019-5 PMC4917585 27397965

[b47] Riley J. *et al.* Tracking bees with harmonic radar. Nature 379, 29–30 (1996).8538737

[b48] Schmid-Hempel P. Parasites in social insects. (Princeton University Press, 1998).

[b49] McMahon D. P. *et al.* A sting in the spit: widespread cross‐infection of multiple RNA viruses across wild and managed bees. Journal of Animal Ecology 84, 615–624 (2015). 25646973 10.1111/1365-2656.12345 PMC4832299

[b50] Blanchard P. *et al.* Evaluation of a real-time two-step RT-PCR assay for quantitation of Chronic bee paralysis virus (CBPV) genome in experimentally-infected bee tissues and in life stages of a symptomatic colony. Journal of Virological Methods 141, 7–13 (2007). 17166598 10.1016/j.jviromet.2006.11.021

[b51] de Miranda J. R. *et al.* Standard methods for virus research in *Apis mellifera*. Journal of Apicultural Research 52 (2013).

[b52] Human H. *et al.* Miscellaneous standard methods for Apis mellifera research. Journal of Apicultural Research & Bee World 52 (2013).

[b53] Fries I. *et al.* Standard methods for *Nosema* research. J. Apicult. Res. 52, 1–28, doi: 10.3896/ibra.1.52.1.14 (2013).

[b54] Edwards A. M. *et al.* Revisiting Lévy flight search patterns of wandering albatrosses, bumblebees and deer. Nature 449, 1044–1048, doi: 10.1038/nature06199 (2007). 17960243

[b55] Clauset A., Shalizi C. R. & Newman M. E. Power-law distributions in empirical data. SIAM review 51, 661–703 (2009).

[b56] Viswanathan G. M., da Luz M. G. E., Raposo E. P. & Stanley H. E. The physics of foraging: an introduction to random searches and biological encounters. (Cambridge University Press: Cambridge, , 2011).

[b57] Reynolds A. Liberating Lévy walk research from the shackles of optimal foraging. Physics of Life Reviews (2015). 10.1016/j.plrev.2015.03.002 25835600

[b58] Reynolds A. M. *et al.* Evidence for a pervasive ‘idling-mode’activity template in flying and pedestrian insects. Royal Society Open Science 2, 150085 (2015). 26064664 10.1098/rsos.150085 PMC4453252

[b59] Viswanathan G. *et al.* Optimizing the success of random searches. Nature 401, 911–914 (1999). 10553906 10.1038/44831

[b60] Reynolds A. M. Scale-free animal movement patterns: Lévy walks outperform fractional Brownian motions and fractional Lévy motions in random search scenarios. Journal of Physics A -Mathematical and Theoretical 42, 434006, doi: 10.1088/1751-8113/42/43/434006 (2009).

[b61] Higes M. *et al.* How natural infection by *Nosema ceranae* causes honeybee colony collapse. Environmental Microbiology 10, 2659–2669 (2008). 18647336 10.1111/j.1462-2920.2008.01687.x

[b62] Naug D. & Gibbs A. Behavioral changes mediated by hunger in honeybees infected with *Nosema ceranae*. Apidologie 40, 595–599 (2009).

[b63] Kralj J. & Fuchs S. *Nosema sp*. influences flight behavior of infected honeybee (*Apis mellifera*) foragers. Apidologie 41, 21–28, doi: 10.1051/apido/2009046 (2009).

[b64] Dussaubat C. *et al.* Flight behavior and pheromone changes associated to *Nosema ceranae* infection of honey bee workers (*Apis mellifera*) in field conditions. Journal of Invertebrate Pathology 113, 42–51 (2013). 23352958 10.1016/j.jip.2013.01.002

[b65] Neumann P. & Carreck N. L. Honey bee colony losses. Journal of Apicultural Research 49, 1–6 (2010).

[b66] Reynolds A. M., Schultheiss P. & Cheng K. Are Lévy flight patterns derived from the Weber-Fechner law in distance estimation? Behavioral Ecology and Sociobiology 67, 1219–1226, doi: 10.1007/s00265-013-1549-y (2013).

[b67] Reynolds A. M., Bartumeus F., Kölzsch A. & van de Koppel J. Signatures of chaos in animal search patterns. Scientific Reports 6, 23492 (2016). 27019951 10.1038/srep23492 PMC4810431

