# Supplementary material for: Optimal search patterns in honeybee orientation flights are robust against emerging infectious diseases
Source: Sci Rep. 2016 Sep 12;6:32612. doi: 10.1038/srep32612 (PMC5018844; doi:10.1038/srep32612)

Supplementary Information

Title: Optimal search patterns in honeybee orientation flights are robust against emerging infectious diseases

Authors: Stephan Wolf, Elizabeth Nicholls, Andrew M. Reynolds, Patricia Wells, Ka S. Lim, Robert J. Paxton & Juliet L. Osborne

Supplementary Method: Harmonic Radar Tracking

The tracking of a bee's flight relies on a 16 mm long dipole aerial with a Schottky diode, forming a transponder that is vertically attached to the thorax of the bee[1] (Fig. S1a). At ca. 12–15 mg, the weight of the transponder is considerably lighter than a typical nectar or pollen load carried by a bee [2]. The transponder is excited by microwaves emitted from a stationary, horizontally scanning radar system (Fig S1b)(9.41 GHz-transmitter, 3 kHz pulse repetitive frequency) and returns a microwave of a harmonic frequency of the original wave for which the experimental arena is specifically scanned. With the radar system turning at 20 rpm, this provides a positional record of a bee flying within a range of 900 m every 3 seconds [1,3]. The transponder signals are not uniquely identified and only one individual can be tracked at a time. Radar tracking relies on a clear line-of-sight between the radar and the tracked object. Obscuring landscape features like high vegetation, buildings or high terrain may prevent the continuous recording of positional information [4,5]. Based on the recorded radar signals, tracks were manually digitalized using a custom-made TAS - Track Analysis Software V1.0 (by Shane Hatty, Rothamsted Research, 2008) providing range (metres), bearing (radians) and a time stamp for the transponder signal received with each radar revolution (3 sec). From these datasets we directly inferred track duration and pauses (s), track length (m), mean flight speed (ms^{-1}) and maximum displacement distance (i.e. furthest position of a bee from the colony, m). Based on the smallest polygon enclosing the entire track (hull) we inferred track area A (m^2), track perimeter C (m) and calculated isoperimetry I, i.e. overall track circularity:

$$I = 4\pi \frac{A}{C^2}$$

References

1 Riley, J. *et al.* Tracking bees with harmonic radar. *Nature* **379**, 29-30 (1996).

2 Capaldi, E. A. *et al.* Ontogeny of orientation flight in the honeybee revealed by harmonic radar. *Nature* **403**, 537-540 (2000).

3 Riley, J. & Smith, A. Design considerations for an harmonic radar to investigate the flight of insects at low altitude. *Comput. Electron. Agric.* **35**, 151-169 (2002).

4 Osborne, J. L. *et al.* A landscape-scale study of bumble bee foraging range and constancy, using harmonic radar. *Journal of Applied Ecology* **36**, 519-533, doi:10.1046/j.1365-2664.1999.00428.x (1999).

5 Wolf, S. *et al.* So near and yet so far: harmonic radar reveals reduced homing ability of *Nosema* infected honeybees. *PLoS One* **9**, e103989, doi:10.1371/journal.pone.0103989 (2014).

Supplementary Tables and Figures

Figure S1

a) Honeybee worker marked on its thorax with a white number tag upon which a transponder is attached. b) Harmonic Radar Unit with large transmitting dish and smaller receiving dish mounted on a turntable for horizontal scanning at 20 revolutions per minute. The vehicle contains the operating and recording unit. Note that this picture was taken before the experiment and no oilseed rape was available as foraging resource during the data collection.

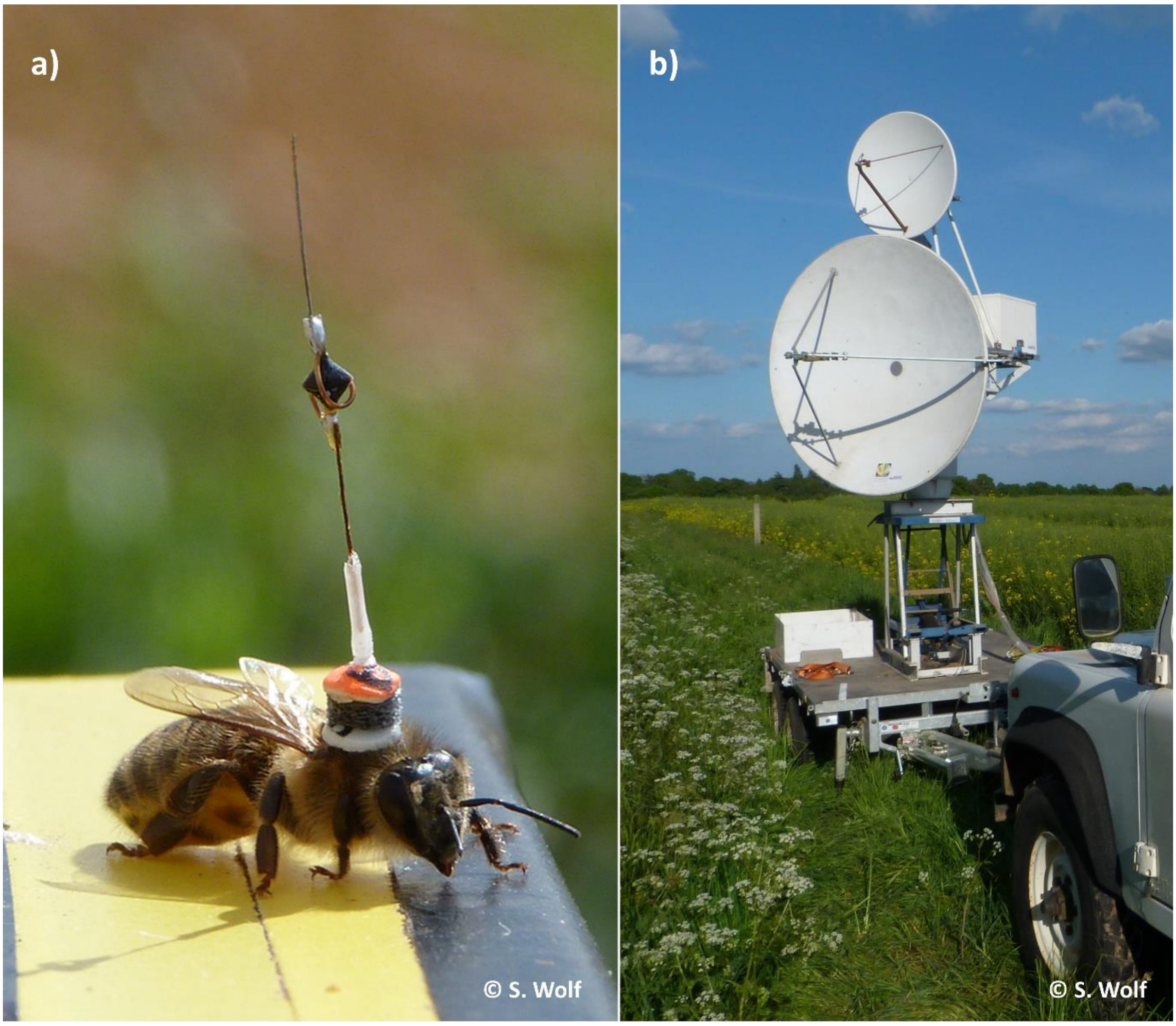

Figure S2

Disease load pyramid for DWV load and *Nosema* spore load in the radar tracked bees. For both pathogens there is a distinct group of non-infected bees, as compared to a group of bees with varying degrees of infections and including highly infected individuals.

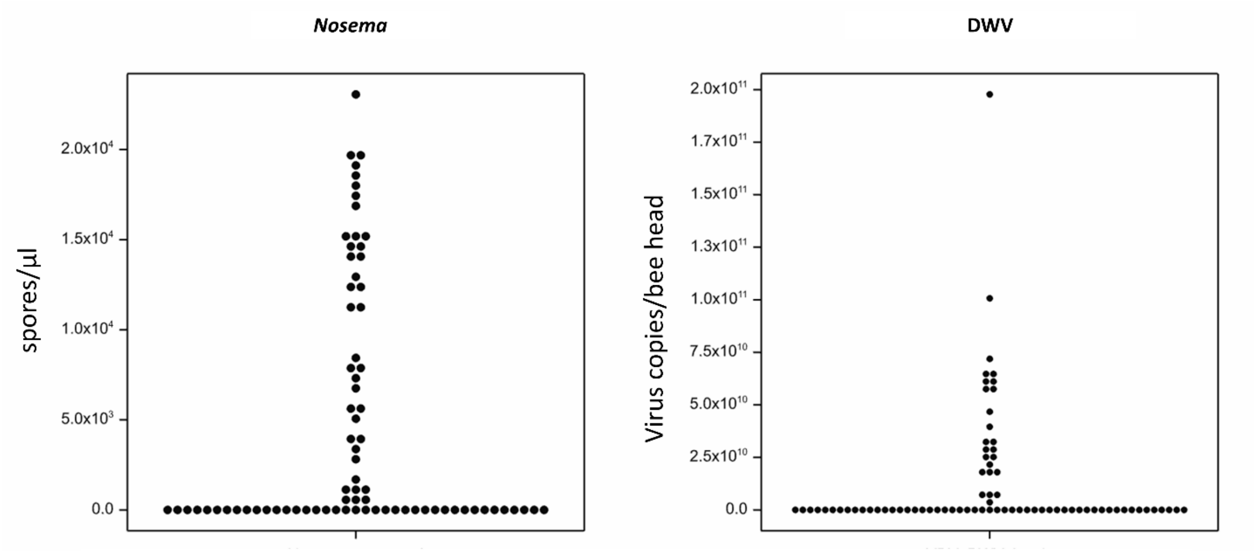

Figure S3

Box plots (box: median (central line) 6 quartiles; whiskers: minimum – maximum values) of the behavioural effects of "high" (>1000 spores/µ, white) and "low" (<1000 spores/µl, light grey) infection levels in honeybee orientation flight parameters in comparison to the absence of *Nosema* sp. ("no", dark grey). See Table 2 for statistical information.

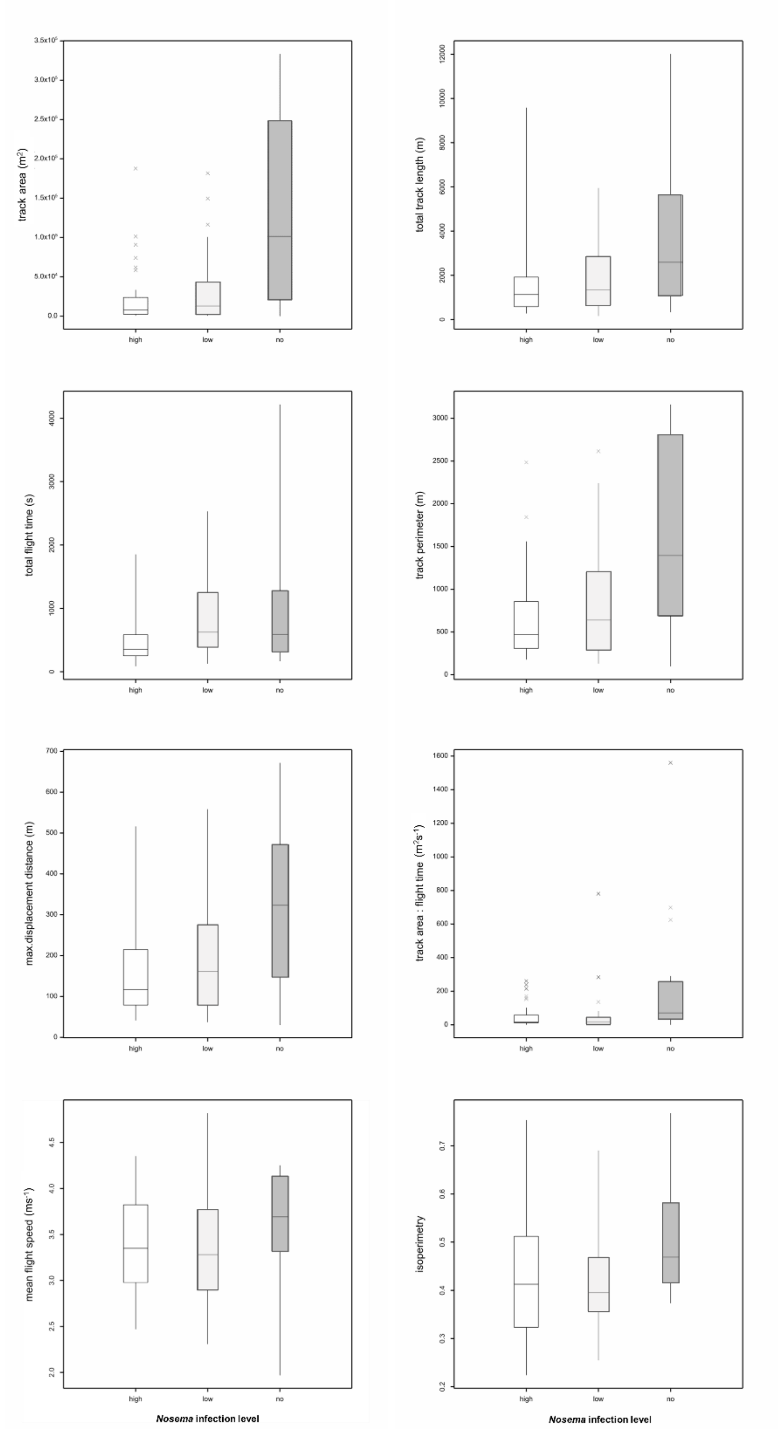

Figure S4

Frequency rank distributions (FRDs) of flight step lengths (l in meters) for all bees in each pathogen group (black solid lines) together with the group-level best-fit power-law distributions (black dashed-line) and the group-level best fit exponential distributions (black dotted lines). For each pathogen group the FRDs are better represented by power-laws which are indicative of Lévy flights.

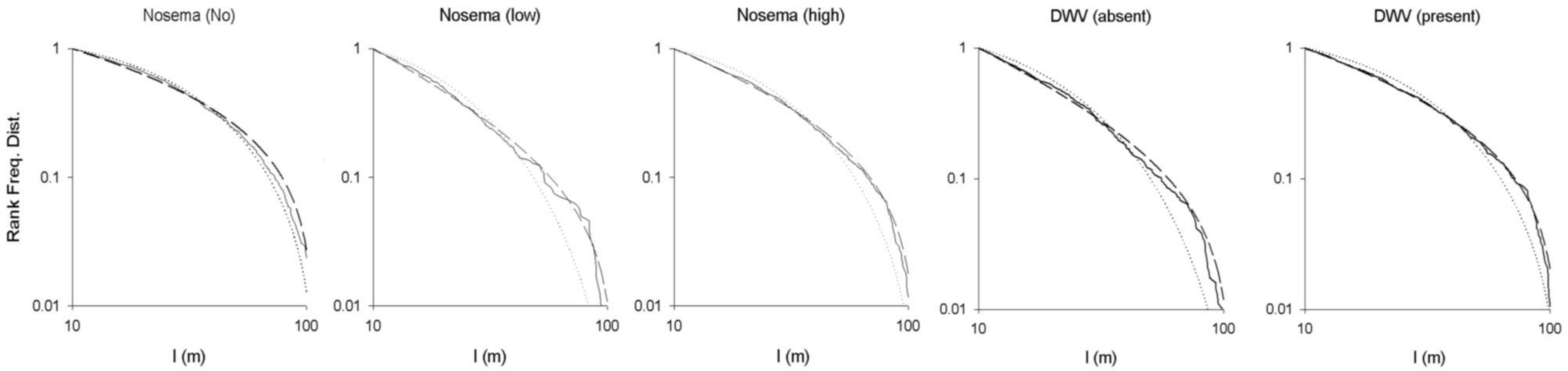

Supplement: Supplementary Information [file srep32612-s1.pdf]
